# Translation of oncology multidisciplinary team meeting (MDM) recommendations into clinical practice

**DOI:** 10.1186/s12913-021-06511-3

**Published:** 2021-05-14

**Authors:** Shalini K. Vinod, Nisali T. Wellege, Sara Kim, Kirsten J. Duggan, Mirette Ibrahim, Jesmin Shafiq

**Affiliations:** 1 Liverpool Cancer Therapy Centre, Liverpool Hospital, Liverpool, NSW Australia; 2 South Western Sydney Clinical School, University of NSW, Liverpool, NSW Australia; 3 Ingham Institute for Applied Medical Research, Liverpool, NSW Australia; 4 South West Sydney Local Health District Clinical Cancer Registry, Liverpool, NSW Australia

**Keywords:** Decision making, Health care quality indicator, Health services research, Hospital oncology service, Patient care team

## Abstract

**Background:**

Multidisciplinary team meeting (MDM) processes differ according to clinical setting and tumour site. This can impact on decision making. This study aimed to evaluate the translation of MDM recommendations into clinical practice across solid tumour MDMs at an academic centre.

**Methods:**

A retrospective audit of oncology records was performed for nine oncology MDMs held at Liverpool Hospital, NSW, Australia from 1/2/17–31/7/17. Information was collected on patient factors (age, gender, country of birth, language, postcode, performance status, comorbidities), tumour factors (diagnosis, stage) and MDM factors (number of MDMs, MDM recommendation). Management was audited up to a year post MDM to record management and identify reasons if discordant with MDM recommendations. Univariate and multivariable regression analyses were performed to assess for factors associated with concordant management.

**Results:**

Eight hundred thirty-five patients were discussed, median age was 65 years and 51.4% were males. 70.8% of patients were presented at first diagnosis, 77% discussed once and treatment recommended in 73.2%. Of 771 patients assessable for concordance, management was fully concordant in 79.4%, partially concordant in 12.8% and discordant in 7.8%. Concordance varied from 84.5% for lung MDM to 97.6% for breast MDMs. On multivariable analysis, breast and upper GI MDMs and discussion at multiple MDMs were significantly associated with concordant management. The most common reason for discordant management was patient/guardian decision (28.3%).

**Conclusion:**

There was variability in translation of MDM recommendations into clinical practice by tumour site. Routine measurement of implementation of MDM recommendations should be considered as a quality indicator of MDM practice.

## Introduction

Multidisciplinary team meetings (MDMs) are a cornerstone of oncology management. MDMs can significantly alter management plans [1–7], are highly likely to recommend care in accordance with clinical practice guidelines [2, 5, 8–11] and increase utilisation rates of treatment [12–14]. MDMs have been documented to improve diagnostic and staging practice, reduce time to treatment and improve survival in some cancers [14–17].

The structure and process of MDMs varies in different clinical settings and for different tumour sites. This can impact on MDM decision making and whether MDM recommendations are implemented into clinical practice [3, 18]. In a systematic review by Lamb et al., MDM decisions could not be implemented in 1–16% of cases often due to patient preferences or comorbidities precluding MDM recommended treatment [3]. An UK study found that MDM recommendations were translated into practice in 91.3% of cases across 14 tumour sites [19]. A German study reported 66% full implementation and 14% partial implementation of MDM decisions across three tumour types [20]. Other studies have shown variable implementation rates of 88% in skin cancer [10], 70–84% in head and neck cancer [6, 20], 80–96% in upper gastrointestinal cancers [13, 21–23], 67–96% in lung cancer [7, 24–26], 97% in thoracic cancers [4], 64–91% in brain cancer [20, 27], 80–90% in colorectal cancer [5, 13, 17, 28], 78% in gynaecological cancer [29], 82–95% in breast cancer [30–33], and 59–73% in sarcomas [20].

There is a paucity of Australian data regarding the clinical translation of MDM recommendations. These are confined to head and neck cancer [6], lung cancer [7], genitourinary cancer [1, 13], gastrointestinal cancer [13] and breast cancer [32, 33]. These are largely small studies of individual tumour site MDMs. The aim of this study was to comprehensively evaluate the translation of MDM recommendations into clinical practice for all solid tumour site MDMs at an Australian academic oncology centre and investigate whether there was significant variation by MDM type. We hypothesised that there would be significant variation in clinical translation of MDM recommendations by MDM type due to the different underlying patient characteristics of different cancer populations and differences in complexity of management. The secondary aim was to identify factors associated with MDM concordant management and identify reasons for discordant management.

## Methods

This was a retrospective audit of oncology and medical records for nine solid tumour site MDMs which occurred between the 1st February and 31st July 2017 at Liverpool Hospital, NSW, Australia. All MDMs were held solely within Liverpool Hospital except for the lung MDM which was videoconferenced with Campbelltown Hospital. These MDMs are attended by all medical specialists involved in diagnosing or treating the particular cancer as well as pathologists, radiologists, nuclear medicine physicians and /or palliative care physicians where relevant. In terms of additional staff, all MDMs are attended by nurse care coordinators, the head and neck MDM by a dietitian and speech pathologist, the upper gastrointestinal MDM by a dietitian and the breast MDM by a geneticist. Electronic meeting agendas recorded in MOSAIQ® were used to identify patients. All patients listed in the MDM agendas were audited and excluded only if there was no evidence of MDM discussion. Ethics approval was obtained for this study from the SWSLHD Human Research Ethics Committee.

All MDMs were electronically documented in either MOSAIQ® or Powerchart™. Five MDMs used a template for recording MDM data either in MOSAIQ® (skin, lung, gynae-oncology) or Powerchart™ (colorectal, upper gastrointestinal). The subheadings used in each template are shown in Table 1. Except for the lung MDM, all data was recorded during the meeting as free text under these subheadings. For the lung MDM, some data was collected in a systematic format before the meeting and the rest was filled in as free text during the meeting. The remaining MDMs used documented MDM discussion and recommendations as free text.

**Table 1:** Standard templates for recording multidisciplinary team meeting (MDM) discussion

Gynae-oncology	Colorectal & Upper GI^a^	Skin	Lung
History of presenting complaint	Type of specialist present	Type of specialist present	Presenting consultant^b^
Past medical history	MDT team	History of presenting complaint	Date of presentation^b^
Radiology review	MDT tumour details	Relevant medical history	ECOG performance status^b^
Pathology review	MDT notes	Radiology review	Weight loss^b^
MDM discussion	MDT consensus	Pathology review	Comorbidities^b^
MDM recommendation	MDT outcomes	Stage	Smoking history^b^
		BFRAF status	Pulmonary function tests^b^
		MDM discussion	Investigations performed^b^
		Management recommendations	Stage^c^
			Radiology review
			Pathology review
			PET review
			MDM discussion
			MDM recommendation

*MDT* multidisciplinary team, *ECOG* Eastern Cooperative Oncology Group, *PET* positron emission tomography

^a^Used the same template

^b^Data recorded pre-meeting, systematically with drop down options

^c^Stage recorded pre-meeting but updated if necessary after MDM discussion

Four researchers extracted data for this study. To ensure consistency, a data collection template was used and a random audit performed by a second investigator of 10% of each MDM data. For patients discussed on multiple occasions, data were collected for all MDMs which occurred during the study period. The data collected included patient factors (age, gender, country of birth, preferred language, residential postcode, Eastern Cooperative Oncology Group (ECOG) performance score, Charlson comorbidity index (CCI) [34]), tumour factors (diagnosis, diagnosis date, stage according to AJCC 7th edition) and MDM factors (date, consensus, treatment intent).

Patient’s culturally and linguistically diverse (CALD) status was categorised into “Non-CALD”, “CALD, English”, “CALD, Non-English” or “Unknown” based on country and birth and language. Non-CALD patients included those born in Australia and in countries (Canada, USA, UK, New Zealand and South Africa) where Australia has received significant numbers of English speaking migrants. CALD patients were all those born elsewhere. Postcode of residence was used to assign the Index of Relative Socio-economic Disadvantage (IRSD) quintile [35]. ECOG performance score was recorded if documented in clinical notes a month either side of the MDM date. CCI was calculated by scoring patient comorbidities [34] and then categorised into groups (None, 1–2 and 3+).

MDM presentation was categorised into “MDM Specific Cancer” if this was related to the tumour site of the MDM, “Other Cancer” if this was a primary cancer different to the MDMs tumour site, “Non-Cancer” if the presentation was not related to cancer and “Unknown” groups. Stage was categorised into four groups for data analysis “0–3”, “4/Malignant central nervous system (CNS) tumour”, “Benign tumours” and “Unknown”.

MDM Consensus was categorised as “No further treatment/Clinical follow up”, “Treatment Recommended” and “Other”. “Treatment recommended” included surgery, radiotherapy, systemic therapy and palliative care. The “other” category included recommendations for further imaging or biopsy or genetic testing. For patients presented at multiple MDMs, an overall MDM consensus and management intent was derived after the last MDM. Management intent was categorised as either “Palliative”, “Curative” or “Unknown” as determined by MDM notes and treatment offered.

To assess concordance of management with MDM recommendation, management was audited up to 1 year after the MDM. Patients were categorised as concordance “not assessable” if there was loss to follow-up (including death) after MDM presentation. For the remaining patients, concordance was grouped into “Fully concordant”, “Partially concordant”, and “Not concordant”. Fully concordant was defined as completion of all MDM recommended management within 1 year of discussion, partially concordant was completion of some but not all recommended management within a year and not concordant was lack of implementation of any MDM recommendations. Where management was not concordant with MDM recommendations, medical records were reviewed to ascertain the reason and categorised as “Patient/Guardian Decision”, “Clinician Decision”, “Comorbidity”, “Change in Stage”, “Change in Performance status”, “Other” (for reasons other than previous labels) or “Unknown”.

Statistical analysis was performed using Excel and IBM® SPSS Statistics, V26 (IBM Corporation, New York, USA). Patients whose concordance was not assessable were excluded from concordance rates calculations. MDM consensus was grouped into concordant (fully or partially) and non-concordant for analyses and the proportions in each group for potential influencing factors were tested for statistical significance through chi-square test. These factors were further tested for their strength of relationship with concordance level using univariate and multivariable logistic regression analyses. The factors tested were age, gender, country of birth, preferred language, CALD status, IRSD quintile, ECOG Performance Status score, CCI score, MDM tumour site, stage, reason for presentation, number of MDMs, management and overall consensus. Those factors with a *p*-value less than 0.2 on univariate analysis were entered into a combined multivariable model to assess their individual effect on concordance in presence of other factors.

## Results

There were 835 patients discussed at the MDMs during the 6 month period (Table 2). The lung, breast and upper gastrointestinal MDMs were busiest whilst the skin and genitourinary MDMs had the fewest patient discussions. Overall median age of patients was 65 years but this varied from 53 years in the neuro-oncology MDM to 70 years in the lung MDM. 25.1% came from a CALD Non-English background, highest for breast MDM patients (35.4%) and lowest for skin MDM patients (6.5%). 54.9% resided in the two lowest socioeconomic quintiles.

**Table 2:** Study population characteristics

Characteristic	Overall *n* = 835	Gynae-onc *n* = 70	Colorectal *n* = 87	GU *n* = 63	Skin *n* = 46	Head&Neck *n* = 90	Lung *n* = 143	Breast *n* = 130	Neuro-onc *n* = 89	Upper GI *n* = 117
Median age (range) y	65 (16–95)	62 (17–88)	64 (21–87)	68 (31–85)	67 (35–95)	67 (24–98)	70 (27–92)	61 (38–94)	53 (16–86)	65 (31–90)
Age group (%)										
< 50 y	148 (17.7)	21 (30.0)	15 (17.2)	9 (14.3)	2 (4.3)	11 (12.2)	7 (4.9)	30 (23.1)	31 (34.8)	22 (18.8)
50–59 y	149 (17.8)	11 (15.7)	20 (23.0)	8 (12.7)	3 (6.5)	13 (14.4)	17 (11.9)	31 (23.8)	25 (28.1)	21 (17.9)
60–69 y	241 (28.9)	15 (21.4)	26 (29.9)	19 (30.2)	19 (41.3)	31 (34.4)	47 (32.9)	35 (26.9)	16 (18.0)	33 (28.2)
70–79 y	194 (23.2)	18 (25.7)	16 (18.4)	20 (31.7)	9 (19.6)	20 (22.2)	47 (32.9)	18 (13.8)	15 (16.9)	31 (26.5)
80+ y	103 (12.3)	5 (7.1)	10 (11.5)	7 (11.1)	13 (28.3)	15 (16.7)	25 (17.5)	16 (12.3)	2 (2.2)	10 (8.5)
Gender (%)										
Males	429 (51.4)	70 (100)	41 (47.1)	6 (9.5)	14 (30.4)	25 (27.8)	53 (37.1)	127 (97.7)	45 (50.6)	48 (41.0)
Females	406 (48.6)	0 (0)	46 (52.9)	57 (90.5)	32 (69.6)	65 (72.2)	90 (62.9)	3 (2.3)	44 (49.4)	69 (59.0)
CALD status (%)										
CALD, English	161 (19.3)	13 (18.6)	15 (17.2)	13 (20.6)	5 (10.9)	16 (17.8)	22 (15.4)	43 (33.1)	15 (16.9)	19 (16.2)
CALD, NESB	210 (25.1)	18 (25.7)	21 (24.1)	16 (25.4)	3 (6.5)	12 (13.3)	38 (26.6)	46 (35.4)	25 (28.1)	31 (26.5)
Non-CALD	427 (51.1)	37 (52.9)	41 (47.1)	25 (39.7)	38 (82.6)	62 (68.9)	83 (58.0)	41 (31.5)	47 (52.8)	53 (45.3)
Unknown	37 (4.4)	2 (2.9)	10 (11.5)	9 (14.3)	0 (0)	0 (0)	0 (0)	0 (0)	2 (2.2)	14 (12.0)
IRSD Quintiles (%)										
1- Lowest SES	195 (23.4)	13 (18.6)	23 (26.4)	13 (20.6)	7 (15.2)	24 (26.7)	34 (23.8)	42 (32.3)	20 (22.5)	19 (16.4)
2	263 (31.5)	28 (40.0)	18 (20.7)	16 (25.4)	17 (37.0)	29 (32.2)	64 (44.8)	19 (14.6)	30 (33.7)	42 (36.2)
3	237 (28.4)	15 (21.4)	29 (33.3)	20 (31.7)	14 (30.4)	19 (21.1)	24 (16.8)	57 (43.8)	24 (27.0)	35 (30.2)
4	58 (7.0)	3 (4.3)	7 (8.0)	9 (14.3)	1 (2.2)	7 (7.8)	20 (14.0)	2(1.5)	2 (2.2)	7 (6.0)
5 – Highest SES	76 (9.1)	10 (14.3)	8 (9.2)	4 (6.3)	7 (15.2)	11 (12.2)	1 (0.7)	9 (6.9)	13 (14.6)	13 (11.2)
Unknown	5 (0.6)	1 (1.4)	2 (2.3)	1 (1.6)	0 (0)	0 (0)	0 (0)	1(0.8)	0 (0)	0 (0)
ECOG PS										
0	265 (31.7)	8 (11.4)	25 (28.7)	16 (25.4)	24 (52.2)	18 (20.0)	61 (42.7)	67 (51.5)	27 (30.3)	19 (16.2)
1	144 (17.2)	2 (2.9)	10 (11.5)	4 (6.3)	11 (23.9)	7 (7.8)	57 (39.9)	19 (14.6)	23 (25.8)	11 (9.4)
2	48 (5.7)	1 (1.4)	1 (1.1)	2 (3.2)	3 (6.5)	3 (3.3)	19 (13.3)	9 (6.9)	6 (6.7)	4 (3.4)
3	19 (2.3)	0 (0)	0 (0)	1 (1.6)	0 (0)	0 (0)	5 (3.5)	4 (3.1)	6 (6.7)	3 (2.6)
4	4 (0.5)	0 (0)	1(1.1)	0 (0)	1 (2.2)	1 (1.1)	1 (0.7)	0 (0)	0 (0)	0 (0)
Unknown	355 (42.5)	59 (84.3)	50 (57.5)	40 (63.5)	7 15.2)	61 (67.8)	0 (0.0)	31 (23.8)	27 (30.3)	80 (68.4)
CCI (%)										
0	403 (48.3)	41 (58.6)	57 (65.5)	30 (47.6)	17 (37.0)	33 (36.7)	29 (20.3)	74 (56.9)	61 (68.5)	61 (52.1)
1 to 2	324 (38.8)	26 (37.1)	27 (31.0)	24 (38.1)	20 (43.5)	40 (44.4)	80 (55.9)	42 (32.3)	22 (24.7)	43 (36.8)
3+	102 (12.2)	3 (4.3)	2 (2.3)	7 (11.1)	9 (19.5)	14 (15.6)	34 (23.8)	14 (10.8)	6 (6.8)	13 (11.1)
Unknown	6 (0.7)	0 (0)	1 (1.2)	2 (3.2)	0 (0)	3 (3.3)	0 (0)	0 (0)	0 (0)	0 (0)
Presentation by tumour type (%)										
MDM specific tumour	737 (88.3)	53 (75.7)	84 (96.6)	51 (81.0)	46 (100.0)	79 (87.8)	127 (88.8)	130 (100)	84(94.4)	83 (70.9)
Other cancer	49 (5.9)	4 (5.7)	0 (0)	6 (9.5)	0 (0)	5 (5.6)	7 (4.9)	0 (0)	5 (5.6)	22 (18.8)
Non-cancer	33 (4.0)	13 (18.6)	3 (3.4)	2 (3.2)	0 (0)	6 (6.7)	0 (0.0)	0 (0)	0 (0)	9 (7.7)
Unknown	16 (1.9)	0 (0)	0 (0)	4 (6.3)	0 (0)	0 (0)	9 (6.3)	0 (0)	0 (0)	3 (2.6)
Stage (%)										
0–3	373 (44.7)	34 (48.6)	40 (46.0)	13 (20.6)	36 (78.3)	29 (32.2)	87 (60.8)	111 (85.4)	0 (0.0)	23 (19.7)
4/ Malignant CNS	237 (28.4)	7 (10.0)	20 (23.0)	11 (17.5)	9 (19.6)	41 (45.6)	46 (32.2)	14 (10.8)	57 (64.1)	32 (27.4)
Benign	26 (3.1)	0 (0)	0 (0)	0 (0)	0 (0)	0 (0)	0 (0)	0 (0)	26 (29.2)	0 (0)
Unknown	199 (23.8)	29 (41.4)	27 (31.0)	39 (61.9)	1 (2.2)	20 (22.2)	10 (7.0)	5 (3.8)	6 (6.7)	62 (53.0)
Reason first MDM presentation (%)										
First diagnosis	591 (70.8)	60 (85.7)	53 (60.9)	37 (58.7)	39 (84.8)	62 (68.9)	106 (74.1)	103 (79.2)	50 (56.2)	81 (69.2)
Recurrence	142 (17.0)	7 (10.0)	11 (12.6)	13 (20.6)	6(13.0)	26(28.9)	24 (16.8)	7 (5.4)	34 (38.2)	14 (12.0)
On Treatment	93 (11.1)	3 (4.3)	21 (24.1)	12 (19.0)	1 (2.2)	0 (0)	13 (9.1)	18 (13.9)	3 (3.4)	22 (18.8)
Other	9 (1.1)	0 (0)	2 (2.3)	1 (1.6)	0 (0)	2 (2.2)	0 (0)	2 (1.5)	2 (2.2)	0 (0)
No of MDMs (%)										
1	643 (77)	39 (55.7)	68 (78.2)	58 (92.1	39 (84.8)	65 (72.2)	110 (76.9)	108 (83.1)	69 (77.5)	87 (74.4)
2	166 (19.9)	25 (35.7)	16 (18.4)	5 (7.9)	7 (15.2)	24 (26.7)	30 (21)	19 (14.6)	17 (19.1)	23 (19.7)
3–4	26 (3.1)	6 (8.6)	3 (3.4)	0 (0)	0 (0)	1 (1.1)	3 (2.1)	3 (2.3)	3 (3.4)	7 (5.9)
Overall MDM Consensus (%)										
Treatment	611 (73.2)	55 (78.6)	68 (78.2)	35 (55.6)	32 (69.6)	70 (77.8)	108 (75.5)	116 (89.2)	36 (40.4)	91 (77.8)
No treatment/ clinical follow-up	181 (21.7)	15 (21.4)	12 (13.8)	21 (33.3)	14 (30.4)	18 (20)	25 (17.5)	12 (9.2)	47 (52.8)	17 (14.6)
Other	43 (5.1)	0 (0)	7 (8)	7 (11.1)	0 (0)	2 (2.2)	10 (7)	2 (1.5)	6 (6.7)	9 (7.7)
Overall Intent										
Curative	619 (74.1)	58 (82.9)	59 (67.8)	48 (76.2)	39 (84.8)	72 (80)	73 (51)	117 (90)	70 (78.7)	83 (70.9)
Palliative	203 (24.3)	12 (17.1)	28 (32.2)	15 (23.8)	7 (15.2)	18 (20)	57 (39.9)	13 (10)	19 (21.3)	34 (29.1)
Unknown	13 (1.6)	0 (0)	0 (0)	0 (0)	0 (0)	0 (0)	13 (9.1)	0 (0)	0 (0)	0 (0)

*GU* genitourinary, *GI* gastrointestinal, *CALD* culturally and linguistically diverse, *NESB* Non-English speaking background, *IRSD* index of relative socioeconomic disadvantage, *SES* socioeconomic status, *ECOG PS* Eastern Cooperative Oncology Group performance status, *CCI* Charlson Comorbidity Index

ECOG performance status was unknown in 42.5% of patients and was only well recorded for the lung MDM. 12.2% had a Charlson Comorbidity Index (CCI) of 3 or higher but this varied from 6.8% in the neuro-oncology MDM to 23.8% in the lung MDM. 44.7% of presented cases were Stage 0–3 and 28.4% were either Stage 4 or malignant CNS tumour. Stage 0 comprised 14 patients with in-situ cancers. The head and neck, lung and upper gastrointestinal MDMs had a higher proportion of patients with stage 4 disease. The only discussion of benign pathology was at the neuro-oncology MDM.

88.3% of presentations were for the MDM specific tumour and 70.8% were presented at first diagnosis. 77% of patients were discussed at one MDM and 19.9% at a second MDM. The most common recommendation was for treatment (73.2%). The neuro-oncology MDM was most likely to recommend no further treatment or clinical follow-up. Overall management intent was curative in 74.1%. Palliative management was more likely to be recommended by the lung and colorectal MDMs.

**Fig. 1:**
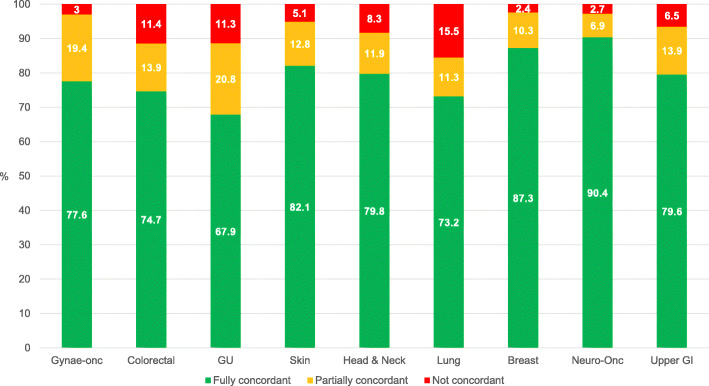
Concordance of clinical management with MDM recommendation by tumour site

For analysis of concordant management with MDM recommendations, 64 patients were excluded due to loss to follow-up (*n* = 50), patient death shortly after MDM (n = 5), no documented management recommendations (*n* = 7) and other reasons (*n* = 2). Of the remaining 771 patients, MDM recommendations were fully translated in 612 (79.4%) patients and partially translated in 99 (12.8%) patients. Clinical management was not concordant with MDM recommendations in 60 (7.8%) patients. Concordance by tumour site MDM is shown in Fig. 1. Full concordance ranged from 67.9% in the genitourinary MDM to 90.4% in the neuro-oncology MDM. Any concordance was equally high for the breast (97.6%), neuro-oncology (97.3%) and gynae-oncology (97%) MDMs and lowest for the lung MDM (84.5%).

The commonest reason for non-concordant management was patient or guardian decision (28.3, 95% CI 18.2–40.4%) (Table 3). In 16.7% (95% CI 9.6–28.4%) there was lack of documentation of implementation of MDM recommendations. Clinician decision was responsible for 10% (95%CI 4.7–20.1%) of discordant reasons, and change in performance status or disease stage, 5% (95% CI 1.7–13.7%) each. Reasons were unable to be ascertained in 20% (95% CI 11.8–31.8%).

**Table 3:** Reasons for discordant management (*n* = 60)

Reason	n	%	95% CI
Patient/guardian decision	17	28.3	18.2–40.4
Lack of documentation	10	16.7	9.6–28.4
Clinician decision	6	10	4.7–20.1
Incomplete/alternative investigation	5	8.3	3.4–17.6
Change in performance status	3	5	1.7–13.7
Change in disease stage	3	5	1.7–13.7
Comorbidity	1	1.7	0.4–9.4
Change in pathology^a^	3	5	1.7–13.7
Unknown reason	12	20	11.8–31.8

^a^2 found to be non-cancer diagnosis, 1 found to be lung metastasis rather than primary

On univariate analysis, patients discussed at breast, neuro-oncology, upper GI and gynae-oncology cancer specific MDMs and had multiple MDMs during the study period showed significantly higher management concordance whereas patients with increasing age, CCI score 3+, unknown presenting diagnosis, unknown stage, unknown treatment intent, and with other consensus had significantly lower concordance (Table 4). After these significant variables were included in a multivariable model, age was no longer significantly associated with management concordance although the odds ratio reduced with increasing age (Table 4). Factors that remained significantly associated with concordance included tumour site, number of MDM discussions and overall consensus. Patients discussed at the breast (OR 5.61, 95% CI 1.51–20.82, *p* = 0.01) and upper gastrointestinal MDMs (OR 2.93, 95% CI 0.99–8.60, *p* = 0.05) had significantly higher concordance with MDM recommendations compared to those discussed at lung MDMs. Patients discussed at multiple MDMs were more likely (OR 2.92, 95% CI 1.17–7.23, *p* = 0.02) to have concordant management. Those whose overall consensus was other, were less likely to have concordant management (OR 0.19, 95% CI 0.07–0.51, *p* = 0.001).

**Table 4:** Univariate and multivariable analyses for management concordance with MDM recommendation

Variable			Univariate analysis			Multivariable analysis		
	Concordant	Not Concordant	Odds ratio (OR)	95% CI for OR	***p***-value	Odds ratio (OR)	95% CI for OR	***p***-value
	N (%)	N (%)		Lower, Upper			Lower, Upper	
Tumour Site					**0.007**			0.148
Lung	120 (84.5)	22 (15.5)	1.000	Reference		1.000	Reference	
Colorectal	70 (88.6)	9 (11.4)	1.426	0.622, 3.269	0.402	1.225	0.458, 3.272	0.686
Genitourinary	47 (88.7)	6 (11.3)	1.436	0.548, 3.764	0.462	1.773	0.573, 5.485	0.320
Skin	37 (94.9)	2 (5.1)	3.392	0.762, 15.105	0.109	2.905	0.615, 13.718	0.178
Head and Neck	77 (91.7)	7 (8.3)	2.017	0.822, 4.947	0.126	1.555	0.567, 4.270	0.391
Breast	123 (97.6)	3 (2.4)	7.517	2.192, 25.773	**0.001**	5.610	1.511, 20.824	**0.010**
Neuro-oncology	71 (97.3)	2 (2.7)	6.508	1.486, 28.504	**0.013**	3.529	0.675, 18.445	0.135
Upper GI	101 (93.5)	7 (6.5)	2.645	1.085, 6.446	**0.032**	2.930	0.998, 8.601	**0.050**
Gynae-oncology	65 (97.0)	2 (3.0)	5.958	1.358, 26.14	**0.018**	3.649	0.721, 18.468	0.118
Age group					0.062			0.372
< 50 years	112 (83.6)	22 (16.4)	1.000	Reference		1.000	Reference	
50–59 years	114 (84.4)	21 (15.6)	0.833	0.248, 2.799	0.768	0.814	0.223, 2.970	0.756
60–69 years	170 (75.9)	54 (24.1)	0.356	0.131, 0.963	**0.042**	0.441	0.146, 1.335	0.147
70–79 years	142 (77.6)	41 (22.4)	0.434	0.154, 1.225	0.115	0.577	0.179, 1.861	0.357
80+ years	74 (77.9)	21 (22.1)	0.268	0.091, 0.789	**0.017**	0.356	0.105, 1.206	0.097
CALD Status					0.393	NI		
CALD, English	117 (78.5)	32 (21.5)	1.000	Reference				
CALD, NESB	151 (77.8)	43 (22.2)	0.859	0.374, 1.969	0.719			
Non-CALD	331 (81.7)	74 (18.3)	0.868	0.415, 1.817	0.707			
Unknown	13 (56.5)	10 (43.5)	0.342	0.097, 1.198	0.093			
IRSD Quintile					0.478	NI		
1 - Lowest SES	150 (82.4)	32 (17.6)	1.000	Reference				
2	191 (77.6)	55 (22.4)	0.935	0.479, 1.824	0.843			
3	168 (77.8)	48 (22.2)	1.505	0.704, 3.218	0.292			
4	46 (82.1)	10 (17.9)	0.983	0.343, 2.815	0.975			
5 - Highest SES	54 (83.1)	11 (16.9)	3.036	0.679, 13.584	0.146			
Unknown	3 (50.0)	3 (50.0)	0.482	0.053, 4.382	0.517			
CCI Score					0.079			0.530
None	291 (79.7)	74 (20.3)	1.000	Reference		1.000	Reference	
1–2	242 (78.6)	66 (21.4)	0.635	0.352, 1.148	0.133	0.917	0.462, 1.817	0.803
3+	79 (80.6)	19 (19.4)	0.438	0.207, 0.924	**0.030**	0.616	0.253, 1.500	0.286
ECOG PS					0.895	NI		
0–2	365 (81.8)	81 (18.2)	1.000	Reference				
3–4	16 (69.6)	7 (30.4)	0.839	0.188, 3.734	0.818			
Unknown	231 (76.5)	71 (23.5)	0.885	0.515, 1.522	0.659			
Stage					**0.010**			0.083
0–3/Benign	298 (79.9)	75 (20.1)	1.000	Reference		1.000	Reference	
4/Malignant CNS	196 (85.2)	34 (14.8)	1.554	0.755, 3.196	0.231	1.824	0.792, 4.199	0.158
Unknown	118 (70.2)	50 (29.8)	0.518	0.286, 0.939	**0.030**	0.611	0.261, 1.428	0.255
MDM Presentation					**0.001**			0.501
MDM Specific Tumour	547 (80.2)	135 (19.8)	1.000	Reference		1.000	Reference	
Other Cancer	40 (85.1)	7 (14.9)	0.796	0.274, 2.312	0.674	0.664		0.487
Not Cancer	18 (69.2)	8 (30.8)	0.567	0.164, 1.959	0.370	1.281		0.751
Unknown	7 (43.8)	9 (56.3)	0.123	0.043, 0.354	**0.000**	0.385		0.195
Reason for MDM 1					0.791	NI		
First diagnosis/ Other	424 (78.7)	115 (21.3)	1.000	Reference				
Recurrence	114 (80.3)	28 (19.7)	0.795	0.413, 1.531	0.493			
During Treatment	74 (82.2)	16 (17.8)	0.950	0.412, 2.193	0.905			
Number of MDMs								
1	468 (80.1)	116 (19.9)	1.000	Reference		1.000	Reference	
2+	144 (77.0)	43 (23.0)	3.074	1.300, 7.264	**0.011**	2.921	1.179, 7.234	**0.021**
Overall Intent					0.146			0.811
Curative	449 (80.5)	109 (19.5)	1.000	Reference		1.000	Reference	
Palliative	153 (76.5)	47 (23.5)	0.912	0.500, 1.665	0.764	0.891	0.440, 1.805	0.749
Unknown	10 (76.9)	3 (23.1)	0.264	0.070, 0.998	**0.050**	1.529	0.294, 7.939	0.614
Overall Consensus					**0.000**			**0.002**
Treatment recommended	468 (79.6)	120 (20.4)	1.000	Reference		1.000	Reference	
No treatment/Clinical follow-up	122 (83.0)	25 (17.0)	0.854	0.426, 1.715	0.658	1.197	0.537, 2.666	0.660
Other	22 (61.1)	14 (38.9)	0.157	0.072, 0.343	**0.000**	0.195	0.075, 0.512	**0.001**

*CNS* Central Nervous System, *CALD* culturally and linguistically diverse, *NESB* Non-English speaking background, *IRSD* index of relative socioeconomic disadvantage, *SES* socioeconomic status, *ECOG PS* Eastern Cooperative Oncology Group performance status, *CCI* Charlson Comorbidity Index, *NI* Not included in multivariable analysis due to univariate *p* value > 0.20

## Discussion

This study provides a comprehensive audit of solid tumour MDM practice at an academic institution. Four MDMs (head and neck, gynae-oncology, neuro-oncology and genitourinary) were held only at Liverpool Hospital. Liverpool Hospital is the sole tertiary referral hospital in South Western Sydney Local Health District providing the only surgical services for head and neck cancers, gynae-oncology and neuro-oncology. Additional MDMs existed at secondary hospitals in SWSLHD for breast cancer (2), upper gastrointestinal cancer (1) and lung cancer (1). The number of patients discussed varied amongst the MDMs. This is partially explained by the varied incidence of cancers and availability of other MDMs. For the major tumour sites, the number of patients discussed at the Liverpool Hospital MDMs represented approximately 14% of genitourinary cancers, 45% of breast cancers, 32% of bowel cancers and 59% of respiratory cancers diagnosed in SWSLHD for a 6 month period [36]. There is an underrepresentation of discussion of genitourinary cases at MDMs, a similar finding to Atwell et al. [37] In Australia, the decision to discuss cases at a MDM is made by the clinician and not mandated by legislation as in France [25] or for cancer society certification as in Germany [20].

Overall there was a high rate of translation of MDM recommendations into clinical practice although this did vary by MDM tumour site. The highest rates of any concordance were seen in the breast and neuro-oncology MDMs. Patient presentations at these MDMs are usually post-operative, as per clinician preference, hence the decision is often about adjuvant therapies only. The younger age of the neuro-oncology patients and lower comorbidity burden in both groups may make it easier to implement recommendations. On multivariable analysis, breast and upper gastrointestinal MDMs were significantly associated with concordant management compared to the lung MDM, although the latter result was of borderline significance. For all the remaining MDMs, odds ratios were greater than one suggesting higher concordance than the lung MDM although this did not reach statistical significance. Lung cancer patients are usually presented de novo where a choice between multiple treatment modalities must be made. In addition these patients had the highest median age and higher rates of comorbidities which impacts on the ability to implement MDM recommendations. Previous analysis in this patient group has shown that although the lung MDM recommends guideline-based treatment in 71% [11] only 54% of patients receive this, largely due to declining performance status, large tumour volumes and comorbidities [8].

Discussion at multiple MDMs was significantly associated with concordant management. This is likely to reflect the collection of further information whether this is patient related (eg assessment of comorbidities or preference) or cancer related (eg imaging or pathology) to better characterise the patient and tailor the MDM recommendation. All the necessary information may not always be present at the first MDM discussion. Goolam-Hossen et al. reported availability of new clinical information in upper GI MDMs as a reason for management change in one third of discordant cases [23]. A survey of clinicians found that a barrier to non-implementation of MDM recommendations was lack of consideration of patient choice or comorbidities [38].

MDM consensus of “other” was significantly associated with lack of concordant management. This category had a small number of patients with diverse MDM recommendations so no firm conclusions can be drawn. Management concordance declined with age although this was not significant on multivariable analysis. Studies of breast MDMs have found older age (> 70–75 years) [31, 33] and younger age (< 35 years) [33] were associated with discordant management. Similarly, concordance declined with increasing CCI score but did not reach statistical significance, suggesting MDMs were taking this into account when making recommendations. Of note, sociodemographic factors such as cultural and linguistic diversity and socioeconomic disadvantage were not associated with translation of MDM recommendations confirming equity of care in a diverse patient population. This may reflect Australia’s universal health care system. In contrast, an American study of lung cancer MDMs found that health insurance status and race were significantly associated with discordant care [24].

Comparisons of management concordance with MDM recommendations in the literature are complicated because of different study populations and use of methodologies (Table 5). Some studies measured only specific MDM recommendations such as adjuvant treatment for breast cancer [32] or radiotherapy for lung cancer [26] whilst others like ours recorded all decisions including biopsy, imaging, treatment, observation and supportive care [4, 7, 17, 24, 27, 28]. Some studies excluded recommendations for investigations, supportive care or management of recurrent disease [23] whilst others included recommendations for clinical trials and genetics referrals [33].

**Table 5:** Comparison of findings with published literature

Author & Country	Study type	Study period	n	Number of MDM decisions	Concordance Rate %- Patients	Concordance Rate % - Decisions
Neuro-oncology						
Hollunder et al., Germany [20]	Retrospective	2014–2016	1406	2176		64-80^a^
Lutterbach et al., Germany [27]	Retrospective	1998–2003	500	–	91	–
Current Study	Retrospective	2017	73	–	90 – 97^a^	
Breast						
English et al., England [31]	Prospective	2007	210	289	_	93
Pattanasri et al., Australia [32]	Retrospective	2010 & 2014	375	–	82	–
Rajan et al., England [30]	Retrospective	2009–2011	705	3230	_	92
Samarasinghe et al., Australia [33]	Retrospective	2011–2016	925	2599	92	96
Current Study	Retrospective	2017	126	–	87 – 97^a^	
Colorectal						
Au-Yeung et al., Australia [13]	Retrospective	2010	37	–	89	
Alfarhan et al., Saudi Arabia [5]	Retrospective	2013	104^b^	158	–	87
Munro et al., Scotland [17]	Retrospective	2006–2007	513	–	80	–
Wood et al., England [28]	Prospective	2005–2006	157	201	_	90
Current Study	Retrospective	2017	79	–	75 – 89^a^	
Upper Gastrointestinal						
Au-Yeung et al., Australia [13]	Retrospective	2010	37	–	92	
Blazeby et al., England [21]	Prospective	2003–2004	273	271	–	85
Bumm et al., Germany [22]	Retrospective	1999–2006	730	807		96
Goolam-Hossen et al., England [23]	Retrospective	2006	363	–	81	–
Gashin et al., USA [39]	Retrospective	2009–2010	137^c^	419	34	65
Current study	Retrospective	2017	108	–	80 – 94^a^	
Genitourinary						
Au-Yeung et al., Australia [13]	Retrospective	2010	86	–	99	
Current Study	Retrospective	2017	53	–	68–89	
Gynaecological						
Current Study	Retrospective		67	–	78 – 97^a^	
Head and Neck						
Brunner et al., Australia [6]	Prospective	2011–2012	158	–	84	–
Hollunder et al., Germany [20]	Retrospective	2014–2016	812	1319		70-82^a^
Current Study	Retrospective	2017	84		80 – 92^a^	
Lung						
Leo et al., France [25]	Prospective	2003–2004	344	–	96	–
Loh et al., New Zealand [26]	Retrospective	2009	110	–	71	–
Osarogiagbon et al., USA [24]	Retrospective	2006–2009	376	454	63	62
Ung et al., Australia [7]	Prospective	2011	65	–	72	–
Current Study	Retrospective	2017	142	–	73 – 85^a^	–
Skin						
Caudron et al., France [10]	Retrospective	2006–2007	228	309	–	80 – 88^a^
Current Study	Retrospective	2017	39	–	82 – 95^a^	
Multiple Tumour Sites						
De leso et al., England [19]	Retrospective	2010	551^d^	–	91	–
Hollunder et al., Germany [20]	Retrospective	2014–2016	2450^e^	3815		66-80^a^
Schmidt et al., USA [4]	Prospective	2010–2012	587^f^	843	98^g^	–
Current Study	Retrospective	2017	771		79 – 92^a^	–

^a^Lowest figure is “fully concordant’ and highest figure is ‘fully and partially concordant’

^b^Included all GI cancers but majority (63%) colorectal

^c^Only included hepatocellular carcinoma

^d^Included 14 adult cancer MDMs

^e^Included neurooncology, head and neck cancers and musculoskeletal tumours

^f^Included lung and oesophagus cancers

^g^Concordance data only available in 52% of patients treated at single institution

This study reports concordance rates per patient. Other studies have reported concordance rates either per patient, per decision or both (Table 5). Reporting concordance per MDM decision will identify if there are specific management recommendations that are not being implemented. Conversely, reporting concordance per patient enables us to gain an overview of overall patient management allowing for multifaceted recommendations which are common. However, the downside of this is management will be considered partially concordant if any recommendations such as further investigations are not implemented.

We chose to report both full and partial concordance with MDM recommendations as management decisions often involve multiple recommendations and may involve multiple MDM presentations. Only Caudron et al. [10] and Hollunder et al. [20] also report full and partial concordance for MDM recommendations. Others have explicitly defined concordance as having all aspects of MDM recommendation implemented [7, 30]. However it is unclear how the majority of studies approach this as they report concordant or discordant translation of MDM recommendations often after just the first MDM. Despite all these caveats, our rates of MDM recommendation concordance with clinical practice are in keeping with published literature across all tumour sites.

The commonest reason for non-concordant management was patient or guardian decision seen in 28.3% similar to the 31–36% reported in the other multiple MDM studies [19, 20]. Patient choice is a common reason for non-concordant management in breast MDMs ranging from 42 to 71%, usually in relation to the acceptance of adjuvant therapy after surgery [21, 30, 32]. In other cancer MDMs patient decision is responsible for discordant management in 11–54% in lung cancer [7, 24, 25], 18–34% in upper gastrointestinal cancers [21, 23], 44% in skin cancer [10], 45% in head and neck cancers [20], 33% in neurological tumours [20] and 14% in sarcomas [20]. Clinician decision not to implement MDM recommendations comprised 10% of reasons, less than the 23–24% reported by the other multiple MDM studies [19, 20]. In other studies this varies from 20% for breast [31], 30% for liver cancer [39] and 8–61% for lung MDMs [24, 25]. A possible reason for these differences is that it is sometimes hard to separate pure clinician decision from other causes such as patient’s poor performance status and comorbidity, which may have been included encompassed in clinician decision as a reason.

We found comorbidity as cause on discordant management in only 1.7% of cases, unlike other studies which reported this as being a major factor for certain tumour types. De Ieso et al. found comorbidity to be a reason for discordance in 33% of cases [19] whilst it was not listed as a reason at all by Hollunder et al. [20], perhaps being incorporated into either patient or clinician decision. It is a major reason for discordance in upper gastrointestinal MDM management (42–45%) [21, 23, 28]. It is sometimes included as clinician decision [24] or combined with patient deterioration [19]. The low impact of comorbidity is a surprising finding. It may be that MDMs take patient comorbidities into account when making management recommendations or that this factor may have been poorly recorded as a decision making factor in patients notes, and may be part of the 20% unknown reasons in our study. The unknown reasons could not be ascertained due to lack of documentation and are likely to represent patient or clinician preference, or comorbidities precluding MDM recommended management.

Rather than identifying specific reasons, a better approach may be to classify justifiable and unjustifiable reasons for discordant management [30]. Patients preference, performance status and comorbidities would all be considered justifiable whilst clinician decision with no other reason or unknown reason would be considered unjustifiable. Measuring the clinical translation of MDM recommendations should be considered a quality indicator for MDMs. Given the vast differences in healthcare settings and patient populations, benchmarks are not appropriate. However, identifying and investigating those who received discordant care is important to ensure the reason is valid and that there is equity of cancer care across different sociodemographic patient populations. A NHS report on effective MDMs states that processes should be in place to ensure MDM recommendations are implemented and that the MDM is notified of any significant changes to the management plan [40]. In Germany, certification of MDMs by the German Cancer Society requires any deviation from MDM recommendations to be documented and assessed [20].

This study is limited by its retrospective nature, with inevitable missing data and inability to ascertain reasons for discordant management in some cases. In addition, Australia’s universal health care system may limit applicability in overseas jurisdictions. The strengths of this study are its large patient cohort and inclusion of all patients discussed at solid tumour MDMs in an academic institution.

Moving forward we plan to perform regular audit and feedback of MDM recommended treatment with treatment received and investigate where there is discordance. However as it will be difficult to report on multifaceted recommendations, these audits will largely be confined to specific anti-cancer treatments ie systemic therapy, radiotherapy and surgery. Since completion of this study, the head and neck, gynae-oncology, CNS and breast MDMs have developed or improved their standardised templates in MOSAIQ® to better record MDM discussion and recommendations in a systematic fashion enabling audit. A template report measuring specific treatment recommendations against treatment received has already been created for the Lung MDM which documents everything including treatment received in MOSAIQ®. This will be more challenging to do for MDMs which document in other electronic medical records and which lack systematic recording of recommendations.

## Conclusion

There was a high rate of translation of MDM recommendations into clinical practice for all solid tumour MDMs in South-Western Sydney although this was variable across tumour sites. Breast and upper gastrointestinal MDMs and multiple MDM discussions were significantly associated with concordant management. Sociodemographic factors did not impact on management concordance. The most common reason for discordant management was patient or guardian decision. Routine measurement of implementation of MDM recommendations should be considered as a quality indicator of MDM practice.

## Data Availability

The datasets generated and/or analysed during the current study are not publicly available as per the ethics committee approval of this study. They are available from the corresponding author on reasonable request after seeking further approval from the South Western Sydney Local Health District Human Research Ethics Committee.
